# Supplementary material for: Electrostatics facilitate midair host attachment in parasitic jumping nematodes
Source: Proc Natl Acad Sci U S A. 2025 Oct 14;122(42):e2503555122. doi: 10.1073/pnas.2503555122 (PMC12557510; doi:10.1073/pnas.2503555122)
Supplement: Supplementary file 1 — Appendix 01 (PDF) [file pnas.2503555122.sapp.pdf]

PNAS

Supporting Information for

Electrostatics facilitate mid-air host attachment in parasitic jumping nematodes

Ranjiangshang Ran, Justin C. Burton, Sunny Kumar, Saad Bhamla, Adler R. Dillman, and Victor M. Ortega-Jimenez

Victor M. Ortega-Jimenez.
E-mail: vortex@berkeley.edu

Justin C. Burton.
E-mail: justin.c.burton@emory.edu

This PDF file includes:

Supporting text
Figs. S1 to S11
Table S1
Legends for Movies S1 to S9
SI References

Other supporting materials for this manuscript include the following:

Movies S1 to S9

Supporting Information Text

Experimental Setup

Figure S1(a) shows the experimental setup for electrostatic experiments. Water drops containing living *Steinernema carpocapsae* nematodes were deposited on a vertically orientated wet filter paper sheet. The wet filter paper was folded back and forth to have a thickness of greater than 1 mm ($\approx$ 50 times the nematode thickness) and was connected to the ground through a metal stand. A fruit fly was tethered to a copper wire and connected to a high-voltage power supply (ES5P-10W, Gamma High Voltage Research, Inc.). The voltage on the tethered fly was adjusted from $100-700$ V relative to the ground. The grounded paper was placed at two different heights below the fly, 5.1 mm and 6.2 mm, respectively. Analogous electrostatic experiments were performed by replacing the fruit fly by a charged metal sphere with a diameter of 2.54 mm. Figure S1(b) shows the experimental setup for nematodes drifting in wind. Nematodes jumped from the same folded wet filter paper with air flows generated by a wind tunnel. The flow speed was approximately 0.2 m/s. In both electrostatic and wind experiments, nematodes with successful jumping were recorded using a high-speed camera (Nova S6, Photron USA, Inc.) at a frame rate of 10,000 frames/s.

Takeoff and landing in still air

As a control, we recorded nematodes jumping in still air, without wind and electrostatic effects. Due to their submillimeter size, nematodes have a small Reynolds number ($Re \sim 10^0$) and experience significantly larger air drag forces compared to inertial forces. Consequently, their horizontal velocity during takeoff is greatly damped by air drag upon landing, resulting in an inclined takeoff and a vertical landing, as shown in Figs. S2(a) and (b). This observation is consistent with our numerical simulations that model the nematodes as spheres (main text, Fig. 2C). However, a vertical landing is not always the case. Nematodes experience a drag force perpendicular to their elongated bodies. As a result, the landing angle can vary depending on their body orientation. Figure S2(c) shows an example of a nematode with an inclined landing despite having a vertical takeoff, due to reorientation of its body. It has been suggested that *S. carpocapsae* have the ability of ariel righting (i.e., the ability to reorient themselves in midair), by maintaining a curved body posture to stop their rotations during landing (1). This stop of body rotation is observed in Figs. S2(a), (b), and (c). Nonetheless, further research is needed to better understand whether nematodes can utilize their aerial righting ability to achieve directional landing.

Trajectory Fitting in Three Dimensions

Since electrostatic forces depend on the three-dimensional (3D) distance between nematodes and hosts, we fit the experimental trajectories of jumping nematodes to our theoretical model in three dimensions. Figures S3(a) and S4(a) show the 3D fitting results and experimental data for nematodes' trajectories attracted by a fruit fly and by a charged metal ball, respectively. The two-dimensional (2D) projection of these 3D trajectories are shown in Figs. S3(b) and S4(b), respectively. For both the fruit fly and the metal ball data, the nematodes' trajectories exhibit considerable out-of-plane displacement and velocity components, indicating the necessity of 3D fitting. Figure S5(a) shows the experimental data for a nematode's displacement, $x(t)$ and $y(t)$, as a function of time (red and blue symbols), and their model fitting results (solid black curves). Figure S5(b) shows the 2D displacement in space, $\mathbf{x} = x\hat{\mathbf{x}} + y\hat{\mathbf{y}}$, for both experimental data (red dots) and model fitting (black curves). We notice that the model fitting results capture the nematode's displacement extraordinarily well, both as a function of time and in space. Figure S5(c) shows the 3D displacement of the nematode, $\mathbf{x} = x\hat{\mathbf{x}} + y\hat{\mathbf{y}} + z\hat{\mathbf{z}}$, with the inferred the out-of-plane displacement, $z(t)$. This illustrates the difference between the full 3D displacement (black curves), the 2D displacement (blue curves), and the experimental data (red dots).

Markov chain Monte Carlo method

Our theoretical model [Eq. (1), main text] has a set of 6 fitting parameters: $\mathbf{\Pi} = [q,\, a_h,\, u_0,\, v_0,\, w_0,\, z_0]$. Here, q and a_h are the charge and the hydrodynamic radius of a jumping nematode, u_0, v_0, w_0 are the three components of the nematode's initial or jumping velocity: $\mathbf{U}_0 = u_0\hat{\mathbf{x}} + v_0\hat{\mathbf{y}} + w_0\hat{\mathbf{z}}$, and z_0 is the out-of-plane component of the nematode's initial position. We want to identify a plausible range of fitting parameters, $\mathbf{\Pi}$, and the unknown data noise level, σ, given the observed data $\mathbf{X} = [x,\, y]$, where x and y are the experimental measurement of the nematode's position. Statistically, this corresponds to the posterior probability distribution $P(\mathbf{\Pi}, \sigma|\mathbf{X})$, which can be computed using Bayes' theorem:

$$P(\mathbf{\Pi}, \sigma|\mathbf{X}) = \frac{P(\mathbf{X}|\mathbf{\Pi}, \sigma)P(\mathbf{\Pi}, \sigma)}{P(\mathbf{X})}, \qquad [1]$$

where $P(\mathbf{X}) = \int P(\mathbf{X}|\mathbf{\Pi}, \sigma)P(\mathbf{\Pi}, \sigma)\, d\mathbf{\Pi}\, d\sigma$ is a normalizing constant (2). We specify a joint prior probability distribution $P(\mathbf{\Pi}, \sigma) = P(\mathbf{\Pi})P(\sigma)$, assuming prior independence between $\mathbf{\Pi}$ and σ. The marginal prior probability $P(\mathbf{\Pi})$ is assumed to be a multivariate normal distribution $\mathbf{\Pi} \sim \mathcal{N}(\mathbf{\Pi}_0, \sigma_0^2\mathbf{A})$, where the factor $\sigma_0^2 = 10$, and $\mathbf{A} = \mathrm{diag}\left(q_s^2,\, a_s^2,\, u_s^2,\, v_s^2,\, w_s^2,\, z_s^2\right)$ is a diagonal matrix such that each component of $\mathbf{\Pi}$ is assumed to be independent. Here, we chose $q_s = 1$ pC, $a_s = 10$ µm, $u_s = v_s = w_s = 0.1$ m/s, and $z_s = 1$ mm. Explicitly, these correspond to prior variances of 10 pC^2, 1000 µm^2, 0.1 m^2/s^2, 0.1 m^2/s^2, 0.1 m^2/s^2, and 10 mm^2, for each parameter in $\mathbf{\Pi}$, respectively. The mean of the prior distribution is chosen to be $\mathbf{\Pi}_0 = \left[q^{(0)},\, a_h^{(0)},\, u_0^{(0)},\, v_0^{(0)},\, w_0^{(0)},\, z_0^{(0)}\right]$, where $q^{(0)} = 0.1$ pC, $a_h^{(0)} = 100$ µm, $w_0^{(0)} = 0$ m/s, and $z_0^{(0)} = 0$ m. Here, $u_0^{(0)}$ and $v_0^{(0)}$

are directly measured from experimental trajectories using a linear least-squares fit of the first 20 data points as a function of time (see blue dashed lines in Fig. S5(a)), which vary for each worm's trajectory. We note that this choice of priors is weakly informative, as discussed in a Section later. The marginal prior distribution $P(\mathbf{\Pi})$ is specified as:

$$P(\mathbf{\Pi}) = \left(\frac{1}{\sqrt{2\pi\sigma_0^2}}\right)^m \det(\mathbf{A})^{-1/2} \exp\left(-\frac{1}{2\sigma_0^2}(\mathbf{\Pi}-\mathbf{\Pi}_0)^{\mathrm{T}}\mathbf{A}^{-1}(\mathbf{\Pi}-\mathbf{\Pi}_0)\right), \quad [2]$$

where $m = 6$ for the six model parameters in $\mathbf{\Pi}$. To ensure positivity of σ, the marginal prior distribution $P(\sigma)$ is assumed to be log-normal, $\sigma \sim \text{Lognormal}(\mu, \tau^2)$, where we specify $\mu = 0$ and $\tau = 4$. This prior allows σ to vary by nearly seven orders of magnitude within its 95% credible interval, and is therefore considered to be weakly informative (3). Here, σ is measured in millimeters, and the prior median, $\exp(\mu)$, corresponds to 1 mm. Explicitly, we have:

$$P(\sigma) = \frac{1}{\sigma\sqrt{2\pi\tau^2}} \exp\left(-\frac{(\log\sigma-\mu)^2}{2\tau^2}\right). \quad [3]$$

The likelihood function $P(\mathbf{X}|\mathbf{\Pi}, \sigma)$ is defined by assuming that the observed data $\mathbf{X} = \{x_i,\, y_i\}_{i=1}^n$ deviate from the model predictions $\mathbf{X}'(\mathbf{\Pi}) = \{x_i',\, y_i'\}_{i=1}^n$ with independent and identically distributed Gaussian noise, whose standard deviation σ is inferred jointly with the six model parameters in $\mathbf{\Pi}$:

$$P(\mathbf{X}|\mathbf{\Pi}, \sigma) = \left(\frac{1}{\sqrt{2\pi\sigma^2}}\right)^{2n} \exp\left(-\frac{1}{2\sigma^2}\sum_{i=1}^{n}\left[\left(x_i - x_i'\right)^2 + \left(y_i - y_i'\right)^2\right]\right). \quad [4]$$

We sampled from the posterior probability distribution $P(\mathbf{\Pi}, \sigma|\mathbf{X})$ using the Metropolis-Hastings (M-H) algorithm (4, 5), which can generate a Markov chain with the following steps:

Step 0. Initialize the Markov chain at $\mathbf{\Pi} = \mathbf{\Pi}_0$ and $\sigma = \exp(\mu)$.

Step 1. Denote the current state as $\mathbf{\Pi}$ and σ. Propose a new state using a Gaussian random walk: $\mathbf{\Pi}' = \mathbf{\Pi} + \boldsymbol{\delta}$ with $\boldsymbol{\delta} \sim \mathcal{N}(\mathbf{0}, \sigma_{\mathrm{rw}}^2\mathbf{A})$, and $\log\sigma' = \log\sigma + \epsilon$, or equivalently, $\sigma' = \sigma\exp(\epsilon)$ with $\epsilon \sim \mathcal{N}(0, \sigma_{\mathrm{rw}}^2)$. The step size σ_{rw} was tuned during pilot runs to achieve an acceptance rate of around 23%, which is considered optimal for multidimensional M-H algorithm (3). In our practice, we found that the values of σ_{rw} typically ranged from 1×10^{-3} to 5×10^{-3}.

Step 2. Calculate the acceptance ratio α:

$$\alpha = \min\left(1, \frac{P(\mathbf{X}|\mathbf{\Pi}', \sigma')P(\mathbf{\Pi}')P(\sigma')}{P(\mathbf{X}|\mathbf{\Pi}, \sigma)P(\mathbf{\Pi})P(\sigma)}\frac{q(\sigma|\sigma')}{q(\sigma'|\sigma)}\right). \quad [5]$$

Note that the proposal distribution for $\mathbf{\Pi}$ is symmetric, i.e., $q(\mathbf{\Pi}|\mathbf{\Pi}') = q(\mathbf{\Pi}'|\mathbf{\Pi})$. However, the proposal distribution for σ is symmetric in $\log\sigma$, which is asymmetric in σ. Thus, the proposal density ratio, $q(\sigma|\sigma')/q(\sigma'|\sigma) = \sigma'/\sigma$, is included to account for the asymmetry, as prescribed for M-H algorithms with asymmetric proposals (3, 5).

Step 3. Generate a random number u from a uniform distribution $\mathcal{U}(0, 1)$. If $u < \alpha$, accept the new state $\mathbf{\Pi} = \mathbf{\Pi}'$ and $\sigma = \sigma'$; otherwise reject $\mathbf{\Pi}'$ and σ' while keep $\mathbf{\Pi} = \mathbf{\Pi}$ and $\sigma = \sigma$. Return to Step 1.

We repeated the above sampling procedure for a total of 50,000 iterations to construct a Markov chain. The first 25,000 iterations of the Markov chain, referred to as the burn-in or warm-up phase, were discarded (3). We can see that all components of $\mathbf{\Pi}$ fluctuate around their mean values (see Fig. S6) during the next 25,000 iterations, known as the stationary phase. We then used the mean values of $\mathbf{\Pi}$ in the stationary phase as the fitting parameters. The total number of iteration of 50,000 was chosen to ensure the convergence of Markov chains, which was quantified by the Gelman-Rubin statistic (see next Section).

Convergence of Markov chains and the Gelman-Rubin statistic

To test the convergence of the Markov chains, we used four different initial values: 1) $q^{(0)} = 0.1$ pC, $a_h^{(0)} = 100$ µm, 2) $q^{(0)} = 0.1$ pC, $a_h^{(0)} = 200$ µm, 3) $q^{(0)} = 0.3$ pC, $a_h^{(0)} = 100$ µm, and 4) $q^{(0)} = 0.3$ pC, $a_h^{(0)} = 200$ µm. The four initial values were chosen to span the plausible ranges of a_h and q. The initial values for other fitting parameters, $u_0^{(0)}$, $v_0^{(0)}$, $w_0^{(0)}$, and $z_0^{(0)}$, were held constant and set equal to the prior means, with values described in the previous Section. Figures S7(a) and S7(b) show the results of this insensitivity test for representative trajectories from experiments conducted with charged fruit fly and metal ball, respectively. We find that, despite being initialized at the four extreme corners of plausible values, all Markov chains converged to the same region of the parametric phase space, demonstrating insensitivity of convergence to initial values.

Next, we quantified the convergence of the Markov chains using the Gelman-Rubin statistic (6). Suppose we run N Markov chains with different initial values, $x_1^{(i)}, x_2^{(i)} ..., x_N^{(i)}$, each chain with n iterations ($i = 1, 2, ..., n$). Here, we have four initial values and thus $N = 4$. Let $\overline{X_j}$ and s_j^2 be the mean and the variance of the j-th chain, respectively, and $\overline{X}$ be the mean of the means of all chains:

$$\overline{X_j} = \frac{1}{n}\sum_{i=1}^{n} x_j^{(i)}, \quad s_j^2 = \frac{1}{n-1}\sum_{i=1}^{n}\left(x_j^{(i)} - \overline{X_j}\right)^2, \quad \overline{X} = \frac{1}{N}\sum_{j=1}^{N}\overline{X_j}. \quad [6]$$

The within-chain variance (W) and the between-chain variance (B) are defined as:

$$W = \frac{1}{N}\sum_{j=1}^{N} s_j^2, \quad \frac{B}{n} = \frac{1}{N-1}\sum_{j=1}^{N}\left(\overline{X_j} - \overline{X}\right)^2. \qquad [7]$$

The Gelman-Rubin statistic, $\hat{R}$, also referred to as the potential scale reduction factor (3), can be estimated as:

$$\hat{R} = \sqrt{\frac{\hat{V}}{W}}, \qquad [8]$$

where $\hat{V}$ is a weighted average of the within-chain and between-chain variances:

$$\hat{V} = \frac{n-1}{n}W + \frac{B}{n}. \qquad [9]$$

Note that $\hat{R} > 1$ is always true, and $\hat{R} \to 1$ as $n \to \infty$. It was argued that $\hat{R} > 1.2$ for any of the model parameters should indicate non-convergence of the Markov chain (6, 7). In common practice, it has been suggested that values of $\hat{R} < 1.1$ are generally considered acceptable (3). Figures S7(c) and S7(d) show the $\hat{R}$ values as a function of iteration numbers n, which was tested in the range of $n = 5,000$ to $n = 60,000$, with burn-in iterations chosen to be $n/2$ (burn-in phase were not included in the calculation of $\hat{R}$). We find that when $n \geq 50,000$, the $\hat{R}$ values for all model parameters drop below $\hat{R} < 1.1$, indicating convergence of the Markov chain. This convergence criterion of $n \geq 50,000$ is true for both representative trajectories and all other trajectories tested. Therefore, we used 50,000 total iterations and 25,000 burn-in iterations as the standard protocol for fitting all experimental trajectories.

Correlations in the posterior distributions and identifiability

In Bayesian statistics, non-identifiability refers to a situation where different values of parameters yield similar likelihoods for the observed data, making it challenging to distinguish between parameter values. When this happens, one would observe flat or multimodal posterior distributions of model parameters (3). Another possible indicator of non-identifiability is the presence of strong linear correlations between one or more model parameters in the posterior distribution, suggesting difficulty to estimate them independently (8). To detect any potential non-identifiability in our inference procedure, we 1) examine the flatness of the posterior distributions by directly plotting them for the charge q, the hydrodynamic radius a_h, and the jumping speed U_0, where U_0 is a combination of 3 model parameters (initial velocities), $U_0 = \sqrt{u_0^2 + v_0^2 + w_0^2}$; and 2) calculate the Pearson correlation coefficient (PCC) for the above model parameters. The PCC between two random variables X and Y is defined as the covariance of them divided by the product of their standard deviations:

$$\rho(X,Y) = \frac{\text{cov}(X,Y)}{\sigma_X \sigma_Y}. \qquad [10]$$

Figures S8(a) and S8(b) show the joint and marginal posterior distributions of U_0 and q, for two representative trajectories of the charged fly and charged metal ball experiments, respectively. We find that the marginal distributions of U_0 and q are not flat nor multimodal; q and U_0 are weakly correlated with PCC ≈ 0. These results suggest we can identify q and U_0 well and independently in our inference procedure.

Figures S8(c) and S8(d) show the joint and marginal posterior distributions of a_h and q, for the same representative trajectories as above. We find a nonlinear correlation between a_h and q in the joint posterior distributions with PCC values of around 0.5. This correlation is the characteristics of our model—higher a_h values lead to larger air drag forces that need larger electrostatic forces (more charge, or larger q) to balance. Figures S8(e) and S8(f) show a similar correlation between U_0 and a_h, with a PCC of ~ 0.9. This is because larger air drag due to higher a_h can be offset by a larger initial velocity U_0 as well. The posterior standard deviations of q, a_h, and U_0 are of orders 0.01 pC, 1 µm, 0.01 m/s, respectively. These values suggest the posterior distributions are narrow and informative. We therefore believe that the observed correlations do not lead to non-identifiability in our inference procedure. We further comment on the posterior variances in the next Section.

Weakly informative prior distributions

To demonstrate that the employed prior distribution, $P(\mathbf{\Pi})$, is weakly informative, we plot the prior and posterior distributions for direct comparison. Figures S9(a) and S9(b) show the prior and posterior distributions for the charge q, where the prior variances are 10 pC^2 while the posterior variances are $\sim 10^{-4}$ pC^2. Figures S9(c) and S9(d) show the prior and posterior distributions for the hydrodynamic radius a_h, where the prior variances are 1000 µm^2 and the posterior variances are ~ 1 µm^2. Figures S9(e) and S9(f) show the prior and posterior distributions for the jumping velocity U_0, where the prior variances are 0.3 m^2/s^2 while the posterior variances are $\sim 10^{-4}$ m^2/s^2. For all three parameters, the prior distributions are essentially flat compared to the much narrower posterior distributions. This indicates our inference is driven by the likelihood (rather than the priors), and the priors are uninformative and do not affect our results.

Justification of electrostatic model assumptions

In our model, we have assumed that the fruit fly host is an isolated charged sphere, and that the folded wet filter paper can be modeled as a grounded infinite plane. These assumptions simplify the model so that it is computationally feasible to calculate the best fit trajectory of a worm in an iterative procedure. But these assumptions may affect our inference of the nematode charge. The first assumption (isolated sphere) introduces a relatively small error. The relationship between the potential (ϕ) and net charge (Q) on an isolated sphere of radius a is $Q = 4\pi\epsilon_0 a\phi$. In the presence of a grounded plane a distance h below the sphere, the capacitance of the system changes and the relationship can be written as:

$$Q = 4\pi\epsilon_0\phi\left(1 + \frac{a}{2h} + \mathcal{O}\left(\frac{a}{h}\right)^2\right), \qquad [11]$$

where we have assumed $h > a$. In our experiments, $h \approx 6$ mm and $a \approx 1$ mm. Thus, the presence of the plane increases the charge on the host by about 10%, which is small compared to the spread of the inferred charge on the worm, q (see Fig. 3*B*, main text).

The second assumption introduces a larger potential error. As stated, the folded, wet filter paper is approximately 1 mm thick, and was used in the experiments so that the nematodes mostly remained in the depth of field of the imaging system, and could tracked accurately. While 1 mm is much larger than the size of the nematodes, it is comparable to the size of the fruit fly host. In reality, the wet filter paper is more like a wedge with a rounded tip of radius ~ 0.5 mm. Charge tends to accumulate near sharp tips and edges, so we may expect that the surface charge (and thus q) would be larger in magnitude than the expectation from an infinite plane. To estimate the size of this effect, we consider just the rounded portion of the filter paper, and model the edge as an infinitely long, conducting cylinder of radius R. The fruit fly host is positioned at a distance $(R + h)$ above the axis of the cylinder. We are interested in the charge density (σ) on the surface of the cylinder immediately below the host. If we assume the charged host is a point charge, this electrostatic problem can be represented as an infinite sum (9):

$$\sigma = -\frac{Q}{2\pi R^2}\sum_{m=-\infty}^{\infty}\int_0^{\infty}\frac{K_m(k(R+h))}{K_m(kR)}dk, \qquad [12]$$

where K_m is the modified Bessel function of the second kind of order m. In the limit of a very large cylinder ($R \to \infty$), this sum reduces to the result for an infinite plane, $\sigma_p = Q/2\pi h^2$. For $h \approx 6$ mm and $R \approx 1$ mm, the charge density is enhanced by $\sigma/\sigma_p \sim 2.6$.

In reality, charge is not just distributed near the rounded edge of the filter paper, so the true enhancement is likely smaller than a factor of 2.6. However, this enhancement would increase the magnitude of the electric field, and a smaller value of q would be necessary to fit the nematode trajectories. Nevertheless, the spread in the data in Fig. 3*B* in the main text is quite large, and 0.1 pC would still be a reasonable estimate of the nematode charge. Ultimately we choose the simplest model for our computational inference procedure, and an error in the inference of q would not change the conclusion that induction is the electrostatic mechanism that controls the charge on the nematodes.

Numerical Simulations of the Charge on a Conducting Cylinder

We used COMSOL Multiphysics® to simulate the electrostatics of an upright conducting cylinder on a grounded plane, similar a standing nematode on a wet filter paper in our experiments. The electric potential, φ, is determined by Laplace's equation:

$$0 = \nabla^2\varphi. \qquad [13]$$

The electric field, $\mathbf{E}$, is the gradient of the electric potential:

$$\mathbf{E} = -\nabla\varphi. \qquad [14]$$

Figure S10(a) shows the domain of the 2D axisymmetric electrostatic simulation, where a sphere at a electric potential ϕ was located at $y = 6.8$ mm and an image sphere at a potential $-\phi$ is located at $y = -6.8$ mm. The electric potential on the two spheres is adjusted between $\phi = 100-700$ V in different simulations. A cylinder was placed between the two spheres with its bottom surface at $y = 0$. The height of the cylinder was fixed at 400 µm, similar to the length of a nematode. The radius of the cylinder varied between $a_1 = 20-200$ µm in different simulations using a parametric sweep. The symmetry of the two spheres makes $y = 0$ an effective grounded plane; the size of the cylinder is small enough compared to the two spheres so that the symmetry is not affected. To ensure grounding, the electric potential on the bottom surface of the cylinder was set to $\varphi = 0$. Figure S10(b) shows the electric potential and the electric field lines in a simulation of $\phi = 500$ V and $a_1 = 20$ µm. We find that both the electric field and potential are highly symmetry about $y = 0$, except in the vicinity of the cylinder. The 3D illustration of the 2D axisymmetric electric potential is shown in Fig. S10(c).

We calculated the electrostatic charge on the cylinder by integrating the normal component of the electric field over the cylinder's surface:

$$q = \frac{1}{\epsilon_0}\oiint_S (\mathbf{E}\cdot\mathbf{n})\,\mathrm{d}A, \qquad [15]$$

where $\epsilon_0 = 8.854 \times 10^{-12}$ F/m is the vacuum permittivity. As a sanity check, we first simulated the charge on a conducting sphere of different radius a, and compared it to the theoretical prediction by Maxwell. Figure S11(a) shows the simulation results of the charge on a sphere of $a = 100-200$ µm, along with Maxwell's prediction. We find that the simulation results agree extraordinarily well with theoretical prediction, validating our simulations. We proceeded with simulating the charge on a conducting cylinder of a fixed height of 400 µm and varying radius a_1. Figure S11(b) shows the simulation results of the charge on a cylinder of $a_1 = 20-200$ µm. A comparison with Maxwell's prediction reveals that while the charge of a cylinder with $a_1 = 20$ µm is lower that of a sphere with $a = 100$ µm, the charge of a cylinder with $a_1 = 40-200$ µm is comparable to that of a sphere with $a = 100-200$ µm, similar to the inferred charge on nematodes in our experiments. This suggests that the observed nematode charge in our experiments (green shaded area, Fig. S11 and Fig. 3B, main text) is larger than the induced charge on a cylinder of the size of a worm. This difference can be attributed to the factor of 2.6 derived in the previous section, due to the geometry of the grounded wet filter paper.

Numerical Simulations of nematodes drifting in wind with electrostatics

We performed numerical simulation of nematodes drifting in wind with a charged host nearby. In our simulations, the center of a charged sphere of a radius $a = 1$ mm was located at $x = 0$ and $y = 6$ mm. The electric potential on the sphere, ϕ, was adjusted between $\phi = 0-800$ V, with a stepwise increment of 25 V. The charge on the sphere was calculated as: $Q = 4\pi\epsilon_0 a\phi$, where ϵ_0 is the vacuum permittivity. A 40-mm long grounded plate was placed at $y = 0$ with its leading edge at $x = -20$ mm and trailing edge at $x = 20$ mm. In each simulation, nematodes took off from the grounded plate with random initial positions uniformly distributed between $x_0 \in [-20, 20]$ mm. The jumping speed of each nematode was fixed at $U_0 = 1$ m/s with random takeoff angles uniformly distributed between $\theta_0 \in [\pi/4, 3\pi/4]$, or between 45° and 135° with respective to the grounded plate. A horizontal laminar flow, $\mathbf{U}_\text{w} = u\hat{\mathbf{x}} + v\hat{\mathbf{y}}$, was included in the simulations with its components modeled by the Blasius solution:

$$u(x,y) = U_\infty f'(\xi),\ \ v(x,y) = \frac{1}{2}\sqrt{\frac{\nu U_\infty}{x}}\left[\xi f'(\xi) - f(\xi)\right], \qquad [16]$$

where the wind speed U_∞ was adjusted from $0-0.6$ m/s with a stepwise increment of 0.025 m/s, $\nu = 1.562 \times 10^{-5}$ m^2/s is the kinematic viscosity of air, and $\xi = y\sqrt{U_\infty/\nu x}$ is a self-similar dimensionless variable. The function $f(\xi)$ is the solution of the ordinary differential equation:

$$2f''' + f''f = 0, \qquad [17]$$

where the prime denotes differentiation with respect to ξ. The equation of motion for the jumping worm with both wind and electrostatics is now given by:

$$m\ddot{\mathbf{x}} = \frac{-Qq\left(\mathbf{x} - \mathbf{x}_0^+\right)}{4\pi\epsilon_0\left|\mathbf{x} - \mathbf{x}_0^+\right|^3} + \frac{Qq\left(\mathbf{x} - \mathbf{x}_0^-\right)}{4\pi\epsilon_0\left|\mathbf{x} - \mathbf{x}_0^-\right|^3} - 6\pi\eta a_h\left(\dot{\mathbf{x}} - \mathbf{U}_\text{w}\right) + m\mathbf{g}. \qquad [18]$$

In each simulation, numerical integration of Eq. (18) was performed in MATLAB® for 1,000 hypothetical nematodes using the built-in function `ode45`. Each nematode has an identical hydrodynamic radius, $a_h = 100$ µm. The integration time for each nematode's trajectory is 0.5 s. Capture rate is calculated from 1,000 trajectories in each simulation, with "capture" defined as successful attachment to the charged sphere at $x = 0$ and $y = 6$ mm. To reduce the computation cost, we employed an analytical first-order approximation of $f(\xi)$, which is otherwise available only through numerical method (10):

$$f_1(\xi) = \xi - 1.8760 + 1.4564\text{e}^{-\xi} + 1.2956\xi\text{e}^{-\xi} + 0.4196\text{e}^{-2\xi}. \qquad [19]$$

We compared the values of $f_1(\xi)$ and its first derivative $f_1'(\xi)$, to the exact value of $f(\xi)$ and $f'(\xi)$ from numerical method in the Table S1. We find the relative error is within 5% for $f_1(\xi)$ at $\xi > 2$ and within 7% for $f_1'(\xi)$ at $\xi > 1$, validating the analytic approximation.

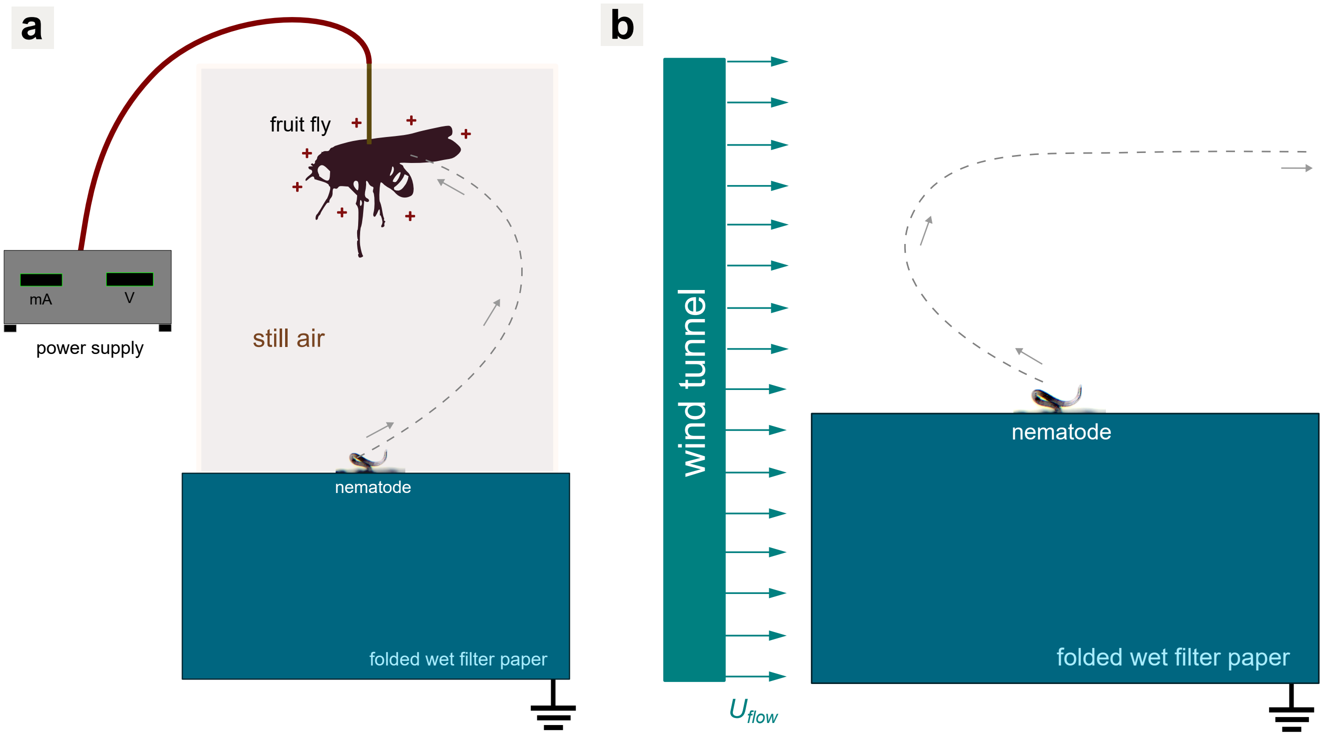

Fig. S1. Schematics of the experimental setup. (*a*) Host attachment of jumping nematodes with electrostatics. Nematodes launched from a vertically oriented sheet of folded wet filter paper to attach to their host, a charged fruit fly, which was tethered to a copper wire and connected to a high-voltage power supply. The wet filter paper was folded back and forth multiple time to have a certain thickness to stand, and was connected to the ground through a metal stand. (*b*) Ariel drifting of jumping nematodes in the wind. Nematodes took off from the same folded wet filter paper without a charged host nearby, and with a wind tunnel generating air flows.

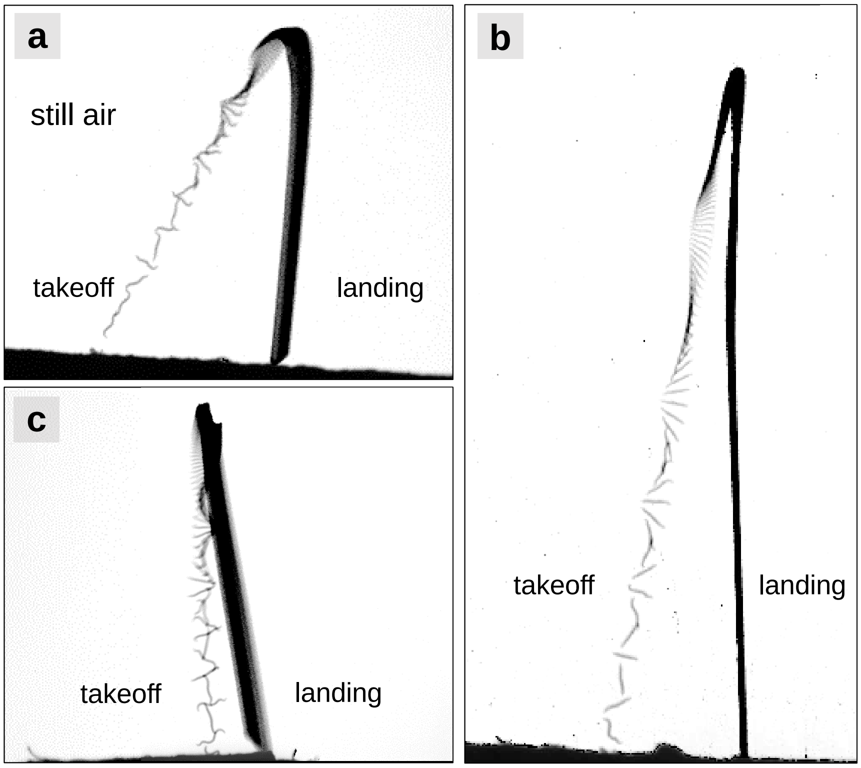

Fig. S2. Takeoff and landing of nematodes jumping in still air. (*a*) A nematode with an inclined takeoff and a vertical landing. (*b*) Another nematode with a inclined takeoff and a vertical landing. (*c*) A nematode with an vertical takeoff and a inclined landing due to body orientation.

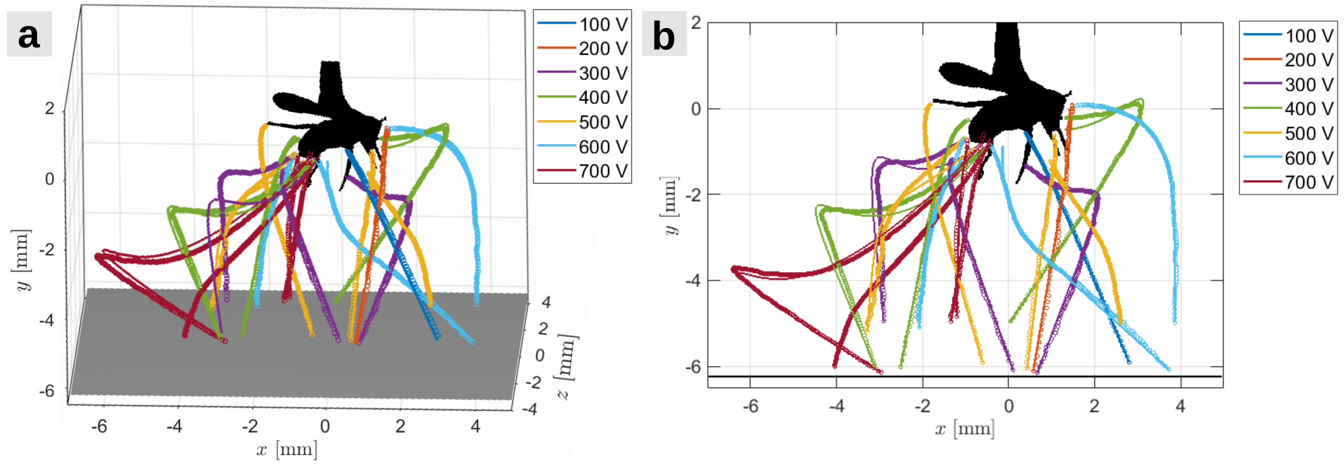

Fig. S3. Jumping nematodes electrostatically attracted by a charged insect host, a fruit fly. (*a*) Three-dimensional (3D) trajectories of the center of mass of jumping nematodes. (*b*) Two-dimensional (2D) projection of the 3D trajectories of jumping nematodes' center of mass. Circle symbols are experimental data, solid curves are model fitting results. Different colors represent different host's electric potentials.

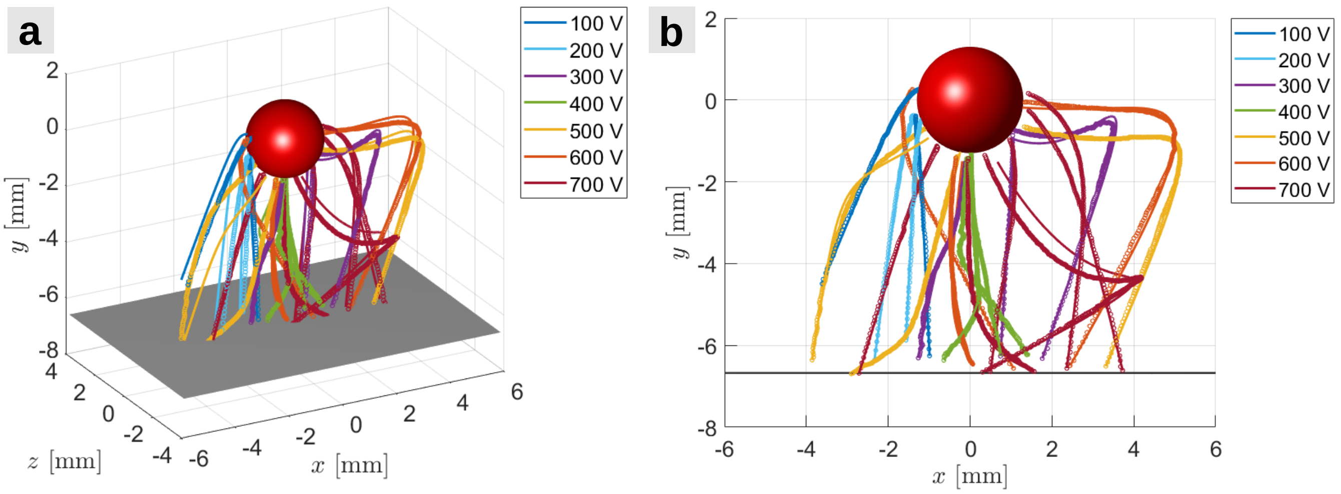

Fig. S4. Jumping nematodes electrostatically attracted by an analogous insect host—a charged metal sphere. (*a*) 3D trajectories of the center of mass of jumping nematodes. (*b*) 2D projection of the 3D trajectories of jumping nematodes' center of mass. Circle symbols are experimental data, solid curves are model fitting results. Different colors represent different host's electric potentials.

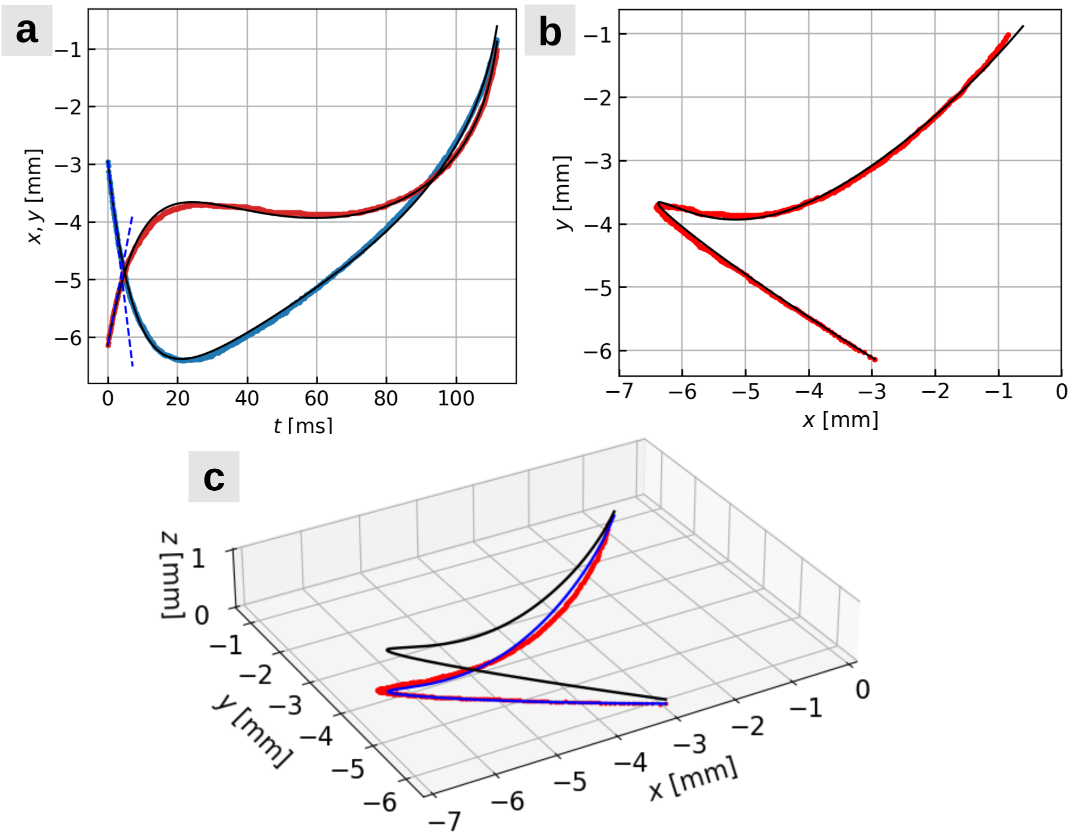

Fig. S5. Model fitting results of a nematode's trajectories with the voltage on the fruit fly at 700 V. (*a*) Experimental data (dots) and model fitting (black curves) for the jumping nematodes' x-position (red) and y-position (blue) as a function of time t. The blue dashed lines represent linear regressions fitted to the first 20 data points, used to estimate the initial velocity. (*b*) Experimental data (red dots) and model fitting (black curves) for the trajectory of the jumping nematode in two dimensions. (*c*) Model fitted 3D trajectory of the jumping nematode (black curves), 2D projection of the 3D trajectory (blue curves), and comparison with the 2D experimental data (red dots).

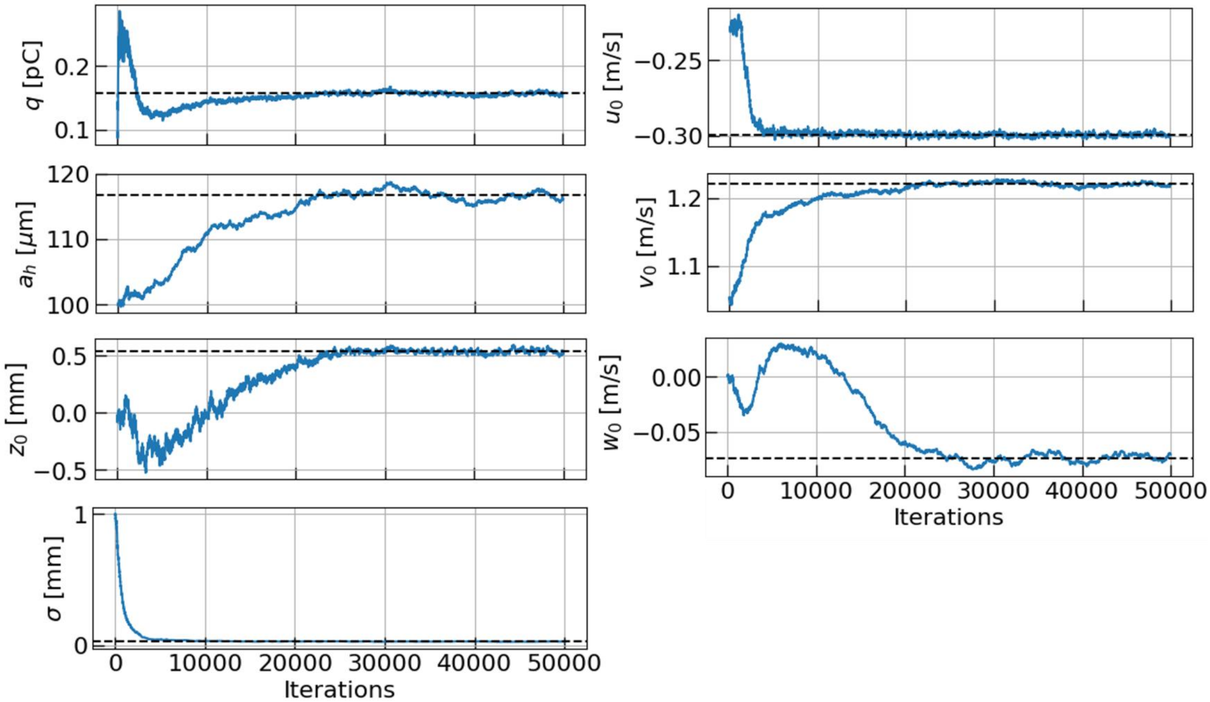

Fig. S6. Evolution of the fitting parameters: q, a_h, z_0, u_0, v_0, w_0, and σ, as a function of number of iterations in a Markov chain, for a total of 50,000 iterations. All parameters reach stationary phase after a "burn-in" phase of 25,000 iterations. The black dashed lines represent the mean values in the stationary phase, taken to be the fitting parameters.

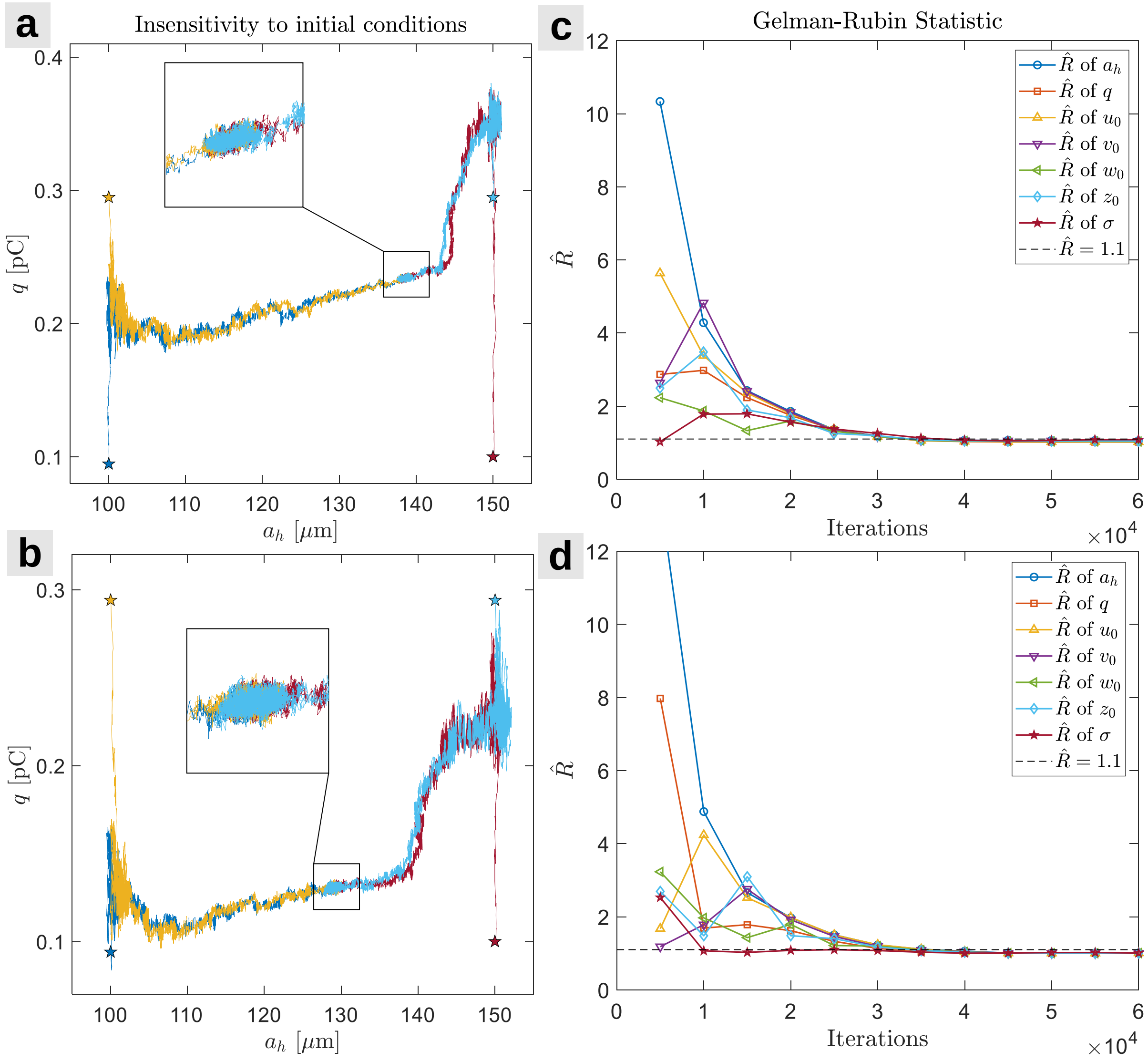

Fig. S7. (*a*, *b*) Insensitivity of Markov chain convergence to initial conditions, for representative trajectories from the experiments conducted with (*a*) the charged fruit fly, and (*b*) the charged metal ball. All 4 initial conditions converge to the same region of the parametric phase space. (*c*, *d*) Gelman-Rubin convergence statistic ($\hat{R}$) as a function of iteration number, for the same representative trajectories as in (*a*) and (*b*), respectively. The $\hat{R}$ values for all fitting parameters drop below $\hat{R} < 1.1$ after 40,000 iterations.

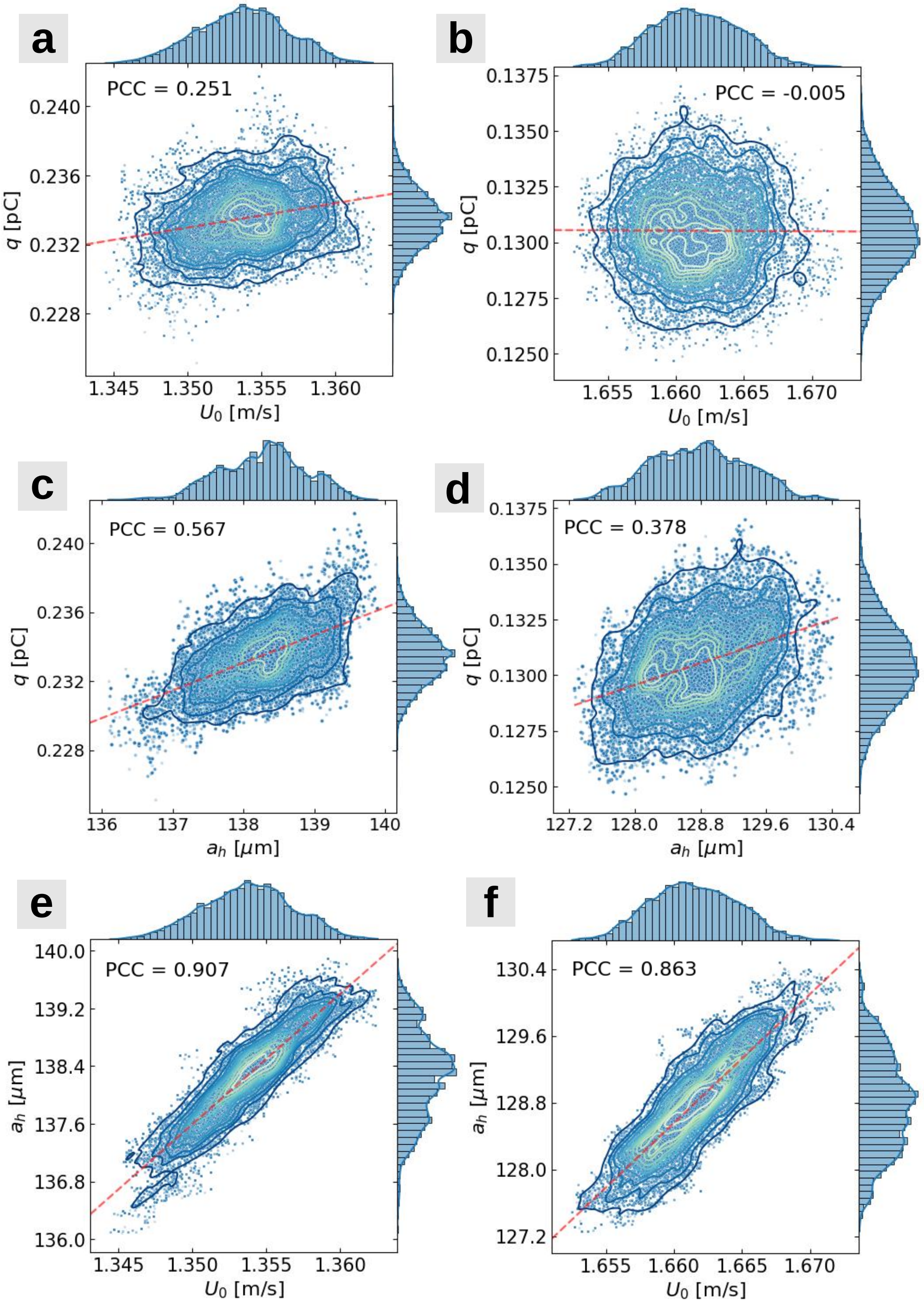

Fig. S8. (*a*, *b*) Joint posterior probability distributions of the jumping speed U_0 and the charge q, for representative trajectories from the experiments conducted with (*a*) the charged fruit fly, and (*b*) the charged metal ball. (*c*, *d*) Joint posterior probability distributions of the hydrodynamic radius a_h and the charge q, for the same representative trajectories as in (*a*) and (*b*), respectively. (*e*, *f*) Joint posterior probability distributions of the jumping speed U_0 and the hydrodynamic radius a_h, for the same representative trajectories as in (*a*) and (*b*), respectively. Here, blue dots are samples from the stationary Markov chain; contours are joint probability density obtained by kernel density estimate (KDE); bar plots are marginal histograms; blue curves are marginal probability density; red dashed lines are linear regressions (95% confidence interval) of the scattered samples; the Pearson correlation coefficients (PCC) are reported in the plots.

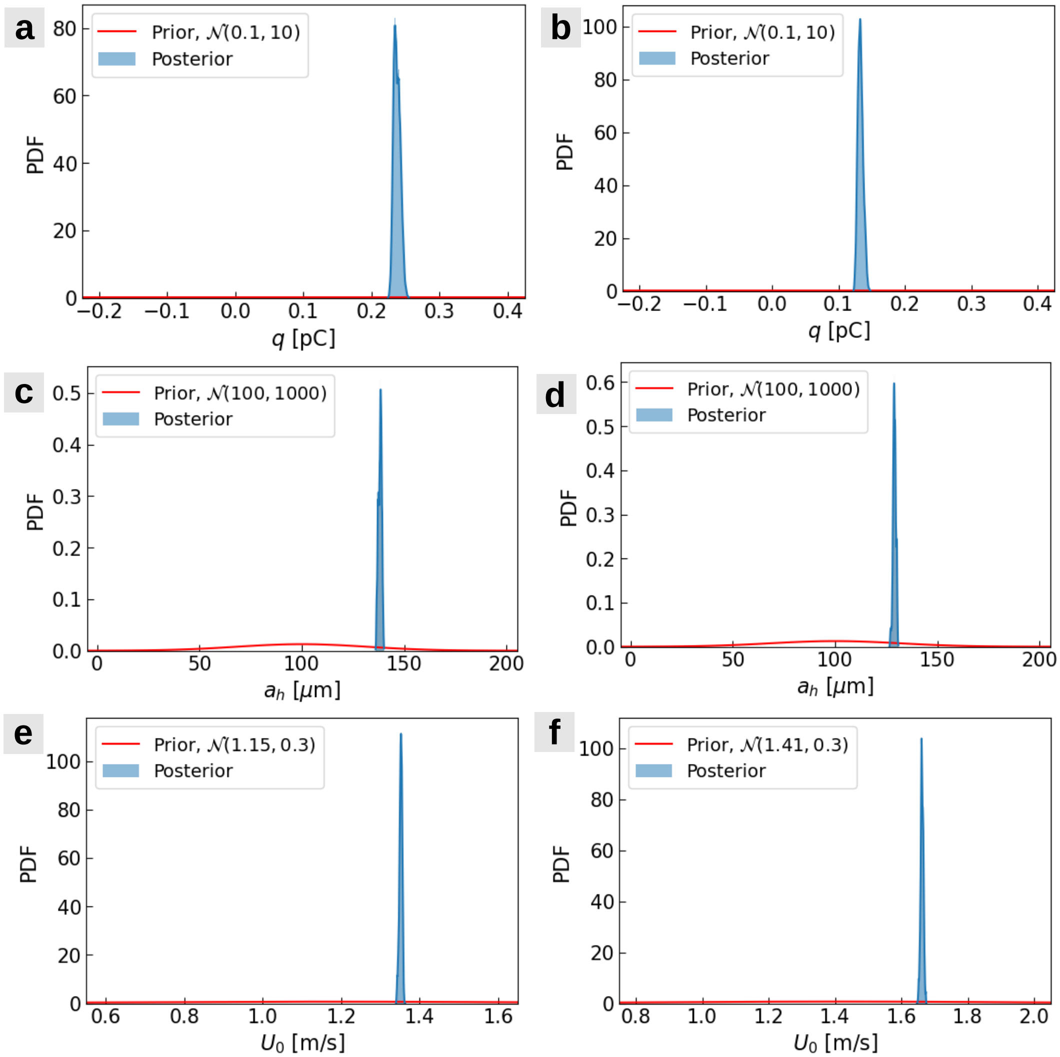

Fig. S9. (*a*, *b*) The prior and posterior distributions of the charge q, for representative trajectories from the experiments conducted with (*a*) the charged fruit fly, and (*b*) the charged metal ball. (*c*, *d*) The prior and posterior distributions of the hydrodynamic radius a_h, for the same representative trajectories as in (*a*) and (*b*), respectively. (*e*, *f*) The prior and posterior distributions of the jumping speed U_0, for the same representative trajectories as in (*a*) and (*b*), respectively. Here, the posterior distributions for all three parameters q, a_h, and U_0 are much narrower than the prior distributions, suggesting the prior distributions are weakly informative.

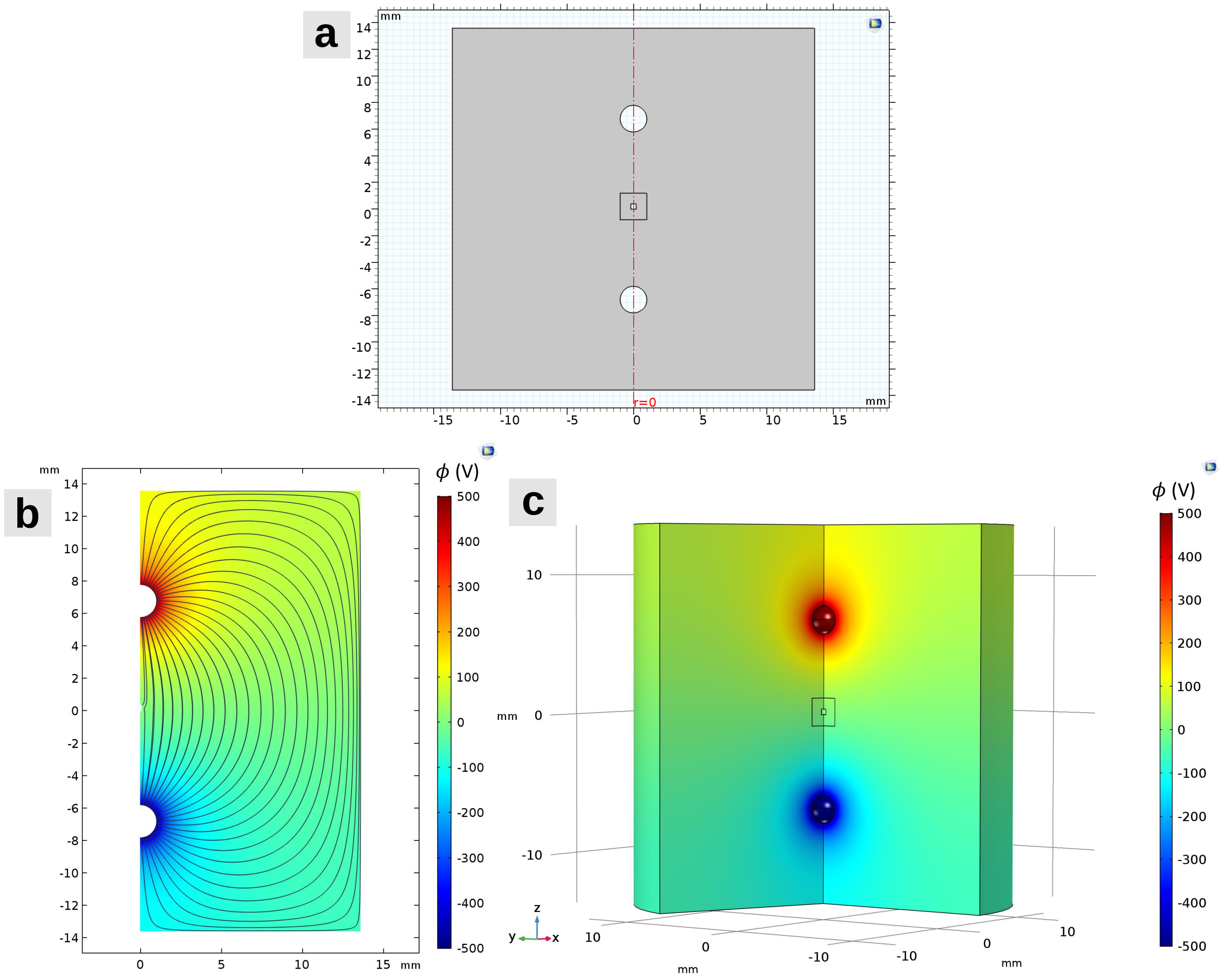

Fig. S10. Numerical simulations of the charge on an upright cylinder using the finite element method in COMSOL Multiphysics®. (*a*) Domain of the 2D axisymmetric electrostatic simulation. Two circles on the top and the bottom are the positively charged sphere and the negatively charged image sphere. A rectangle in the middle is a cylinder of fixed height of 400 μm and a varying radius a_1. The red dashed dotted line in the middle is the axis of symmetry. (*b*) 2D representation of the electric potential and electric field in numerical simulations. Color map represents the electric potential, and black curves are the electric field lines. The voltages on the top and bottom spheres were set to 500 V and -500 V, respectively. (*c*) 3D representation of the same electric potential data presented in (*b*).

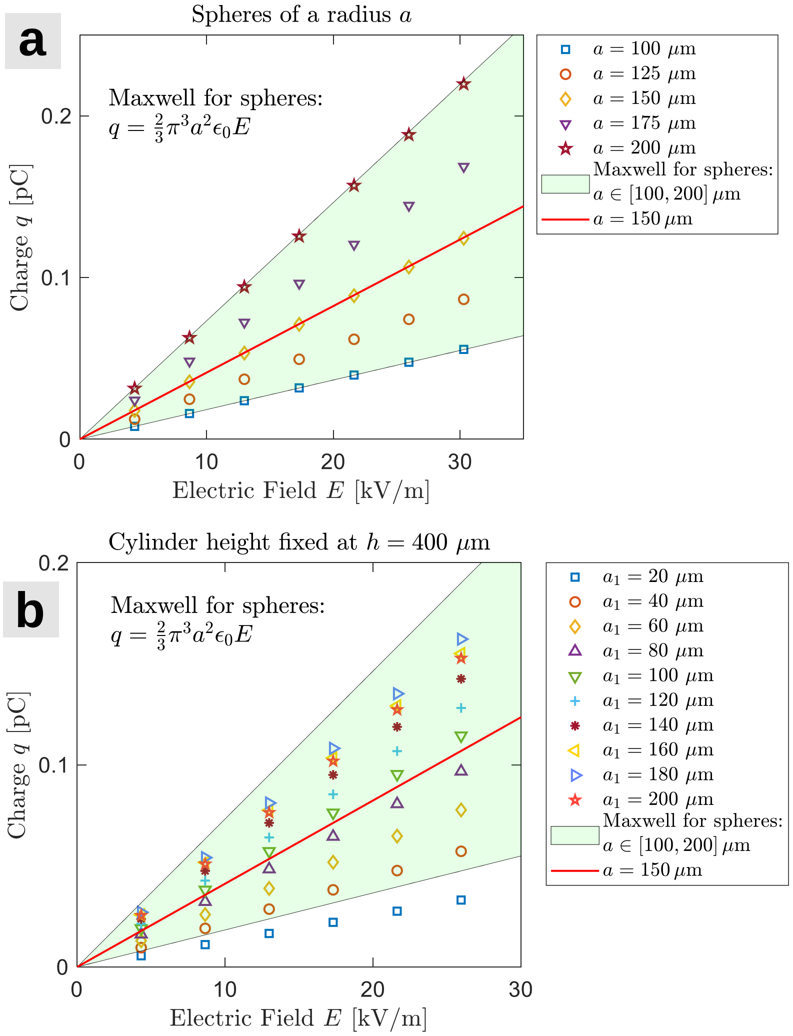

Fig. S11. The induction charge on spheres and cylinders from numerical simulations and comparison with Maxwell's prediction for spheres. (*a*) The induction charge on spheres of a radius $a = 100-200$ μm (markers) and Maxwell's prediction for spheres (shaded area and red line). The numerical data agree almost perfectly with theoretical predictions. (*b*) The induction charge on upright cylinders of a fixed height of 400 μm and varying radius of $a_1 = 20-200$ μm (markers) and Maxwell's prediction for spheres (shaded area). The numerical data for cylinders of radius $a_1 = 40-200$ μm lie within with theoretical predictions for spheres of a radius $a = 100-200$ μm.

Table S1. Comparison between the exact values of the Blasius function $f(\xi)$ and its derivative $f'(\xi)$ from numerical method, and the analytical approximation $f_1(\xi)$ and $f_1'(\xi)$.

ξ	$f(\xi)$	$f_1(\xi)$	error	$f'(\xi)$	$f_1'(\xi)$	error
0	0	0	0	0	0	0
0.5	0.04149	0.05462	31.66%	0.16589	0.20083	21.07%
1.0	0.16557	0.19319	16.68%	0.32979	0.35065	6.32%
1.5	0.37014	0.40349	9.01%	0.48679	0.48871	0.39%
2.0	0.65003	0.67947	4.53%	0.62977	0.61219	2.79%
2.5	0.99631	1.01225	1.60%	0.75126	0.71527	4.79%
3.0	1.39682	1.39106	0.41%	0.84605	0.79640	5.87%
3.5	1.83770	1.80530	1.76%	0.91304	0.85745	6.09%
4.0	2.30576	2.24573	2.60%	0.95552	0.90185	5.62%
4.5	2.79013	2.70500	3.05%	0.97951	0.93334	4.71%
5.0	3.28329	3.17748	3.22%	0.99155	0.95523	3.66%
5.5	3.78057	3.65908	3.21%	0.99688	0.97021	2.68%
6.0	4.27964	4.14688	3.10%	0.99898	0.98033	1.87%
7.0	5.27926	5.13360	2.76%	0.99992	0.99158	0.83%
8.0	6.27923	6.12796	2.41%	1.00000	0.99618	0.18%

Movie S1. Slow motion video (83.3 × slower than real time) of the host attachment process of jumping nematodes in the absence (left) and presence (right) of electrostatic effect. Left: a nematode was unable to attach to a grounded insect host (a fruit fly). Right: a nematode of comparable jumping speed successfully attached a charged host.

Movie S2. Slow motion video (83.3 × slower than real time) of a jumping nematode attracted by a charged insect host (a fruit fly).

Movie S3. Slow motion video (83.3 × slower than real time) of a Jumping nematode attracted by a charged metal sphere, a hypothetical host.

Movie S4. Slow motion video (83.3 × slower than real time) of a jumping nematode drifting in a horizontal laminar flow generated by a wind tunnel.

Movie S5. Numerical simulations of nematodes drifting in wind with a charged spherical host nearby. The host's electric potential (ϕ) was increased from 0 to 800 V, at several fixed wind speeds (U_∞), ranging from 0 to 0.6 m/s. Top shows the rate of successful host attachment (capture rate).

Movie S6. Numerical simulations of nematodes drifting in wind with a charged spherical host nearby. The wind speed (U_∞) was increase from 0 to 0.6 m/s, at several fixed host's electric potentials (ϕ), ranging from 0 to 800 V. Top shows the capture rate.

Movie S7. Slow motion video (333.3 × slower than real time) of a standing nematode attracted by a plastic syringe rubbed on human hair.

Movie S8. Slow motion video (333.3 × slower than real time) of a a group of nematodes attracted by a charged water droplet.

Movie S9. Slow motion video (333.3 × slower than real time) of a falling charged droplet electrostatically attracting and detaching of spores from a grounded surface, with the effect becoming more pronounced as the droplet approaches.

References

1. VM Ortega-Jimenez, et al., Air-to-land transitions: from wingless animals and plant seeds to shuttlecocks and bio-inspired robots. *Bioinspir. Biomim.* **18**, 051001 (2023).
2. P Marjoram, J Molitor, V Plagnol, S Tavaré, Markov chain Monte Carlo without likelihoods. *Proc. Natl. Acad. Sci. U.S.A.* **100**, 15324–15328 (2003).
3. A Gelman, et al., *Bayesian Data Analysis*, Chapman & Hall/CRC Texts in Statistical Science. (Taylor & Francis), 3rd edition, (2013).
4. N Metropolis, AW Rosenbluth, MN Rosenbluth, AH Teller, E Teller, Equation of state calculations by fast computing machines. *J. Chem. Phys.* **21**, 1087–1092 (1953).
5. WK Hastings, Monte Carlo sampling methods using Markov chains and their applications. *Biometrika* **57**, 97–109 (1970).
6. A Gelman, DB Rubin, Inference from Iterative Simulation Using Multiple Sequences. *Stat. Sci.* **7**, 457–472 (1992).
7. SP Brooks, AG and, General methods for monitoring convergence of iterative simulations. *J. Comput. Graph. Stat.* **7**, 434–455 (1998).
8. AE Gelfand, SK Sahu, Identifiability, improper priors, and gibbs sampling for generalized linear models. *J. Am. Stat. Assoc.* **94**, 247–253 (1999).
9. JA Hernandes, AKT Assis, Electric potential due to an infinite conducting cylinder with internal or external point charge. *J. electrostatics* **63**, 1115–1131 (2005).
10. J He, Approximate analytical solution of blasius' equation. *Commun. Nonlinear Sci. Numer. Simul.* **3**, 260–263 (1998).
